# Sulfur Amino Acid Supplementation Abrogates Protective Effects of Caloric Restriction for Enhancing Bone Marrow Regrowth Following Ionizing Radiation

**DOI:** 10.3390/nu14071529

**Published:** 2022-04-06

**Authors:** Christopher Hine, J. Humberto Treviño-Villarreal, Pedro Mejia, Alban Longchamp, Lear E. Brace, Eylul Harputlugil, Sarah J. Mitchell, Jie Yang, Yihong Guan, Jaroslaw P. Maciejewski, Babal K. Jha, James R. Mitchell

**Affiliations:** 1 Department of Cardiovascular and Metabolic Sciences, Cleveland Clinic Lerner Research Institute, Cleveland, OH 44195, USA; yangj3@ccf.org; 2 Department of Molecular Metabolism (Formally Genetics and Complex Diseases), Harvard T.H. Chan School of Public Health, Boston, MA 02115, USA; jhumbertotrevino@gmail.com (J.H.T.-V.) mejia.jp@gmail.com (P.M.); alban.longchamp@chuv.ch (A.L.); learbrace@gmail.com (L.E.B.); eylulharputlugil@gmail.com (E.H.); sarahjayne.mitchell@hest.ethz.ch (S.J.M.); james.mitchell@hest.ethz.ch (J.R.M.); 3 Service of Endocrinology, Department of Internal Medicine, University Hospital and School of Medicine, Universidad Autonoma de Nuevo Leon, Monterrey N.L. 64460, Mexico; 4 Department of Vascular Surgery, Centre Hospitalier Universitaire Vaudois, University of Lausanne, CH-1011 Lausanne, Switzerland; 5 Department of Health Sciences and Technology, ETH Zurich, 8005 Zurich, Switzerland; 6 Department of Translational Hematology & Oncology Research, Taussig Cancer Institute, Cleveland Clinic, Cleveland, OH 44195, USA; yihong_guan@hotmail.com (Y.G.); maciejj@ccf.org (J.P.M.); jhab@ccf.org (B.K.J.)

**Keywords:** caloric restriction (CR), fasting, bone marrow, radioprotection, cystathionine γ-lyase (CGL), hydrogen sulfide (H_2_S)

## Abstract

Radiation therapy damages and depletes total bone marrow (BM) cellularity, compromising safety and limiting effective dosing. Aging also strains total BM and BM hematopoietic stem and progenitor cell (HSPC) renewal and function, resulting in multi-system defects. Interventions that preserve BM and BM HSPC homeostasis thus have potential clinical significance. Here, we report that 50% calorie restriction (CR) for 7-days or fasting for 3-days prior to irradiation improved mouse BM regrowth in the days and weeks post irradiation. Specifically, one week of 50% CR ameliorated loss of total BM cellularity post irradiation compared to *ad libitum*-fed controls. CR-mediated BM protection was abrogated by dietary sulfur amino acid (i.e., cysteine, methionine) supplementation or pharmacological inhibition of sulfur amino acid metabolizing and hydrogen sulfide (H_2_S) producing enzymes. Up to 2-fold increased proliferative capacity of *ex vivo*-irradiated BM isolated from food restricted mice relative to control mice indicates cell autonomy of the protective effect. Pretreatment with H_2_S *in vitro* was sufficient to preserve proliferative capacity by over 50% compared to non-treated cells in *ex vivo*-irradiated BM and BM HSPCs. The exogenous addition of H_2_S inhibited Ten eleven translocation 2 (TET2) activity *in vitro*, thus providing a potential mechanism of action. Short-term CR or fasting therefore offers BM radioprotection and promotes regrowth in part via altered sulfur amino acid metabolism and H_2_S generation, with translational implications for radiation treatment and aging.

## 1. Introduction

More than half of cancer patients receive radiotherapy via γ-rays and X-rays as part of their treatment. Although effective at lessening tumor burden, this ionizing radiation damages and depletes non-cancerous stem and progenitor cells, particularly in the bone marrow (BM), blood, hair follicles, and gut [1,2]. These off-target effects inhibit BM repopulation, hematopoietic stem cell (HSC) self-renewal, cause myelosuppression, promote the development of secondary tumors, and ultimately contribute to poor patient prognosis [3,4]. Importantly, as bone contains a large calcium content, it thus absorbs more irradiation than other tissues and sets up the BM for enhanced damage [5,6]. Severely radiation-damaged bone and BM cause acute as well as latent inflammatory cytokine storms, losses in bone density, and diminished hematopoietic and skeletal stem cell populations, with an inverse increase of adipocytes in the marrow cavity ultimately resulting in BM failure [5,7]. As BM hematopoietic stem cells give rise to cells of myeloid and lymphoid lineages, in the post-irradiation state, BM failure results in systemic immune and hematological disorders, such as aplastic anemia, myelodysplastic and myeloproliferative syndromes, and thrombocytopenia [7]. Thus, interventions to mitigate the side effects of radiation and genotoxic therapies, particularly in the BM, are of clinical importance. 

Aging, like radiation, negatively influences the maintenance, differentiation, and division of BM and BM-derived hematopoietic stem and progenitor cells (HSPCs), leading to biased and/or depleted progenitor and immune cell populations [8]. One of the most effective and well-studied interventional approaches to reduce the rate of aging and increase lifespan is dietary restriction (DR) [9]. DR encompasses a variety of regimens involving reduced total calories, often referred to as calorie restriction (CR), intermittent fasting, and/or specific macronutrient restriction, such as methionine/cysteine restriction (also known as sulfur amino acid; SAA, restriction), without malnutrition for up to an entire lifetime [10]. DR also improves metabolic fitness and increases resistance to many forms of stress even when applied for periods of as little as one week [11,12,13].

While severe malnutrition negatively impacts BM by impairing BM endothelial cell growth, hematopoiesis, and results in anemia and leukopenia [14], long-term DR that does not illicit malnutrition is associated with a number of benefits against aging- and radiation-induced BM deficiencies. For example, 24 months of CR prevents aging-related declines in BM and HSPC-related health in old mice [15], and 9 months of CR starting at 3 months of age prevents skewed myeloid-biased linages and increases HSC repopulation potential when measured in 1-year-old mice [16]. Long-term periodic fasting, a specific form of CR, between 2 to 12 months prevents age-dependent declines in immune cells, platelets, hemoglobin, and red blood cells [17], and protects bone HSCs from genotoxic chemotherapy by promoting proper HSC self-renewal [18]. In addition to delaying aging-related pathologies, long-term periodic fasting for 3 weeks in mice prior to ionizing radiation exposure results in improved femoral bone marrow repopulation and HSPC recovery within one to two weeks post irradiation [19,20], and decreases radiation induced death [21]. Additionally, CR lasting between 4 weeks and the entire adult lifetime prior to, concurrent with, and/or after ionizing radiation exposure reduces the incidence of secondary tumors and improves survival in rodents [22,23]. 

Less is known about the potential of short-term DR to impact radiation-induced BM depletion. However, DR for a week or less protects against other multifactorial stressors including ischemia reperfusion injury to the kidney, liver, and brain in rodents in a manner that is not only dependent on total caloric intake, but also protein and amino acid intake [24,25]. Mechanistically, augmented expression and/or activity of transsulfuration pathway (TSP) enzyme cystathionine γ-lyase (CGL) in response to the intake of dietary sulfur amino acids is implicated in multiple DR benefits, including stress resistance and skeletal muscle angiogenesis [26,27,28]. TSP and CGL are responsible for cysteine biogenesis from methionine, but they also produce hydrogen sulfide (H_2_S) [29], which is implicated in DR-mediated protection against ischemic reperfusion injury (IRI) [30].

Here, we show that short-term CR protects against radiation-induced BM and mixed BM HSPC population damage and promotes regrowth through a mechanism requiring reduced dietary sulfur amino acid intake and downstream H_2_S production enhancement.

## 2. Materials and Methods

### 2.1. Mice

**Figure 1:**
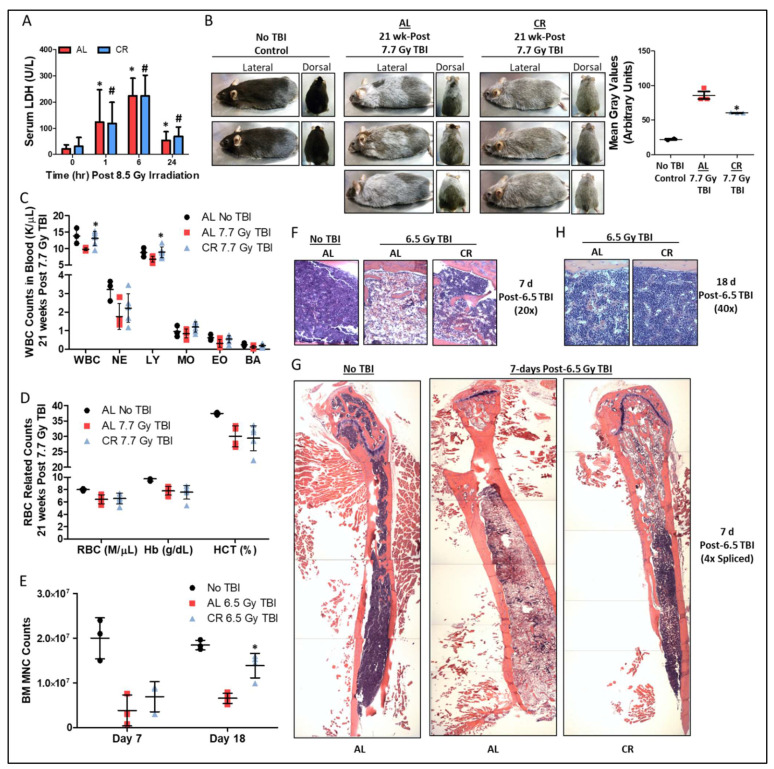
**Short-term CR protects against ionizing radiation induced damage to bone marrow.** (**A**) Serum LDH prior to and up to 24 h after 8.5 Gy TBI in AL and 50% CR fed mice, *n* = 7–11/group. Asterisk indicates the significance of the difference in AL group between time 0 and times 1, 6, and 24, and pound indicates the significance of the difference in CR group between control and times 1, 6, and 24; */^#^ *p* < 0.05. (**B**) Representative lateral and dorsal images and quantification of gray fur at 21-weeks post 7.7 Gy total body irradiation (TBI) in AL or 50% CR preconditioned mice, *n* = 3/group for AL & CR, and *n* = 2/group for No TBI, which serve as qualitative examples of fur color in the absence of TBI. Asterisk indicates the significance of the difference between AL and 50% CR; * *p* < 0.05. (**C**,**D**) Total white blood cell (WBC), neutrophil (NE), lymphocyte (LY), monocyte (MO), eosinophil (EO), and basophil (BA) counts (**C**), as well as red blood cell (RBC) counts, hemoglobin (Hb), and hematocrit (HCT) parameters (**D**) at 21-weeks post 7.7 Gy total body irradiation (TBI) in *ad libitum* (AL) or 50% calorie restricted (CR) preconditioned mice, *n* = 3–6/group. Asterisk indicates the significance of the difference between AL and CR; * *p* < 0.05. (**E**–**H**) Bone marrow mononuclear cell (MNC) counts at day 7 and 18 (**E**), and hind limb bone and bone marrow stained with H&E at day 7 (20× objective, (**F**); 4× objective spliced composites, (**G**)) and day 18 (40× objective, (**H**)) post 6.5 Gy TBI in AL and 50% CR preconditioned mice, *n* = 3–4/group. H&E staining provides a pink stained section (eosin) for the bone/extracellular matrix and occasional attached skeletal muscle, while the purple (hematoxylin) region is the bone marrow (cell nuclei). Lack of the purple (hematoxylin) color is indicative of bone marrow ablation as it is the loss of nucleus containing cellularity, while red blood cells lacking a nucleus will stain pink or red and adipose cells create voids in the purple staining. Asterisk indicates the significance of the difference between AL and 50% CR; * *p* < 0.05. Data are presented as means ± SD.

**Figure 2:**
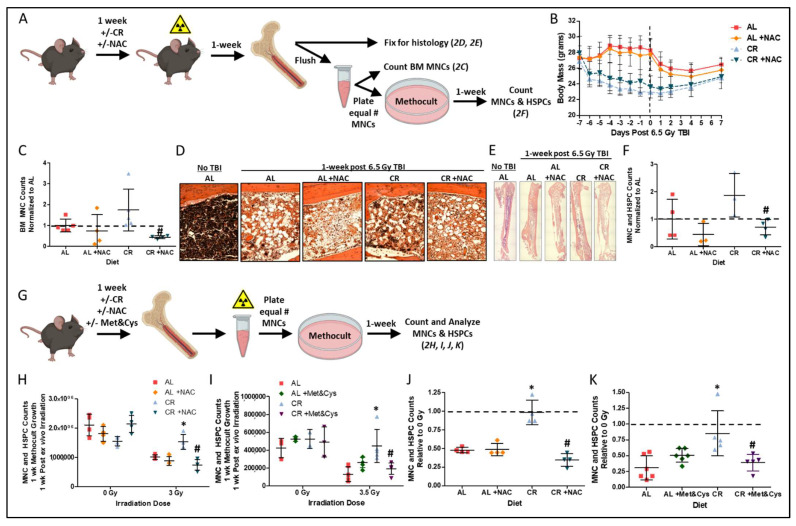
**Sulfur amino acid supplementation suppresses the radio-protective benefits of CR:** (**A**) Schematic for determining *in vivo* and *ex vivo* regeneration potential of bone marrow and bone marrow HSPCs in Methocult after *in vivo* radiation exposure for data shown in B, C, D, E, and F. (**B**) Changes in body mass one week prior to and one week post 6.5 Gy total body irradiation (TBI) in mice preconditioned on AL ± NAC or 50% CR ± NAC diets. Dotted line indicates the day of TBI. (**C**–**E**) Bone marrow mononuclear cell (BM MNC) counts (**C**), and hind limb bone and BM stained with H&E (20× objective, (**D**); 4× objective spliced composites, (**E**) at 1-week post 6.5 Gy TBI in AL ± NAC and 50% CR ± NAC preconditioned mice, *n* = 4–5/group. Pound indicates the significance of the difference between CR and CR+NAC; ^#^ *p* < 0.05. (**F**) MNC and HSPC counts normalized to AL control group after 1-week of Methocult growth, *n* = 3–4 mice/group. Pound indicates the significance of the difference between CR and CR+NAC; ^#^*p* < 0.05. (**G**) Schematic for determining *ex vivo* regeneration potential of BM and BM HSPCs in Methocult after *ex vivo* radiation exposure for data shown in (**F**–**I**). (**H**) Cell counts after 1-week of Methocult growth when mice were preconditioned on AL ± NAC or 50% CR ± NAC diets prior to BM harvest and *ex vivo* 0 Gy or 3 Gy irradiation, *n* = 4 mice/group. Asterisk indicates the significance of the difference between CR and AL, and pound indicates the significance of the difference between CR and CR+NAC; */^#^ *p* < 0.05. (**I**) Cell counts after 1-week of Methocult growth when mice were preconditioned on AL ± Met&Cys or 50% CR ± Met&Cys diets prior to BM harvest and *ex vivo* 0 Gy or 3.5 Gy irradiation, *n* = 3–6 mice/group. Asterisk indicates the significance of the difference between CR and AL, and pound indicates the significance of the difference between CR and CR+Met&Cys; */^#^ *p* < 0.05. (**J**) Cell counts normalized to respective 0 Gy controls as depicted in (**H**) after 1-week of Methocult growth when mice were preconditioned on AL ± NAC or 50% CR ± NAC diets prior to BM harvest and *ex vivo* 3 Gy irradiation, *n* = 4 mice/group. Asterisk indicates the significance of the difference between CR and AL, and pound indicates the significance of the difference between CR and CR+NAC; */^#^ *p* < 0.05. (**K**) Cell counts normalized to respective 0 Gy controls as depicted in (**I**) after 1-week of Methocult growth when mice were preconditioned on AL ± Met&Cys or 50% CR ± Met&Cys diets prior to BM harvest and *ex vivo* 3.5 Gy irradiation, *n* = 3–6 mice/group. Asterisk indicates the significance of the difference between CR and AL, and pound indicates the significance of the difference between CR and CR+Met&Cys; */^#^ *p* < 0.05. Data are presented as means ± SD.

**Figure 3:**
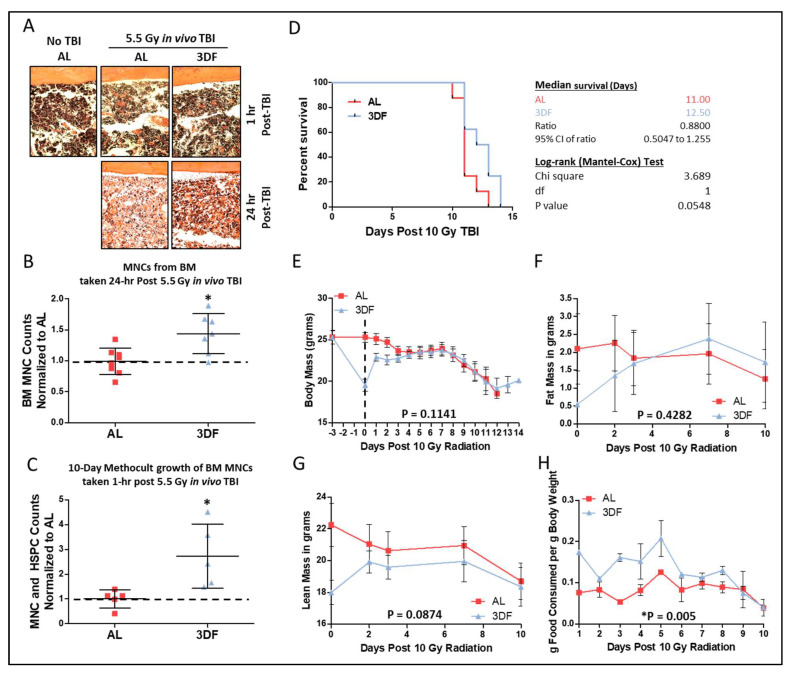
**Fasting preconditioning provides radio-protection for bone marrow and survival.** (**A**) Representative H&E stained femurs (20x objective) harvested 1 h or 24 h post 5.5 Gy TBI from AL or 3-day fasted (3DF) mice. (**B**) Flushed BM MNC counts relative to AL group taken from hindlimbs of AL or 3DF mice 24 h post 5.5 Gy TBI, *n* = 7–8 mice/group. (**C**) 10-day Methocult growth counts of BM MNCs normalized to the AL group originally taken from AL or 3DF mice 1 h post 5.5 Gy TBI, *n* = 5/group. (**D**–**H**) Kaplan–Meier survival plot and associated statistics (**D**), body mass in grams (**E**), fat mass in grams (**F**), lean mass in grams (**G**), and food consumption (**H**) of mice exposed to 10 Gy TBI after being fed AL or 3DF prior to irradiation, *n* = 8 mice/group. Dotted line in (**E**) indicates day of TBI. Asterisks indicate the significance of the difference compared to non-NaHS treatment, * *p* < 0.05. Data are presented as means ± SD.

**Figure 4:**
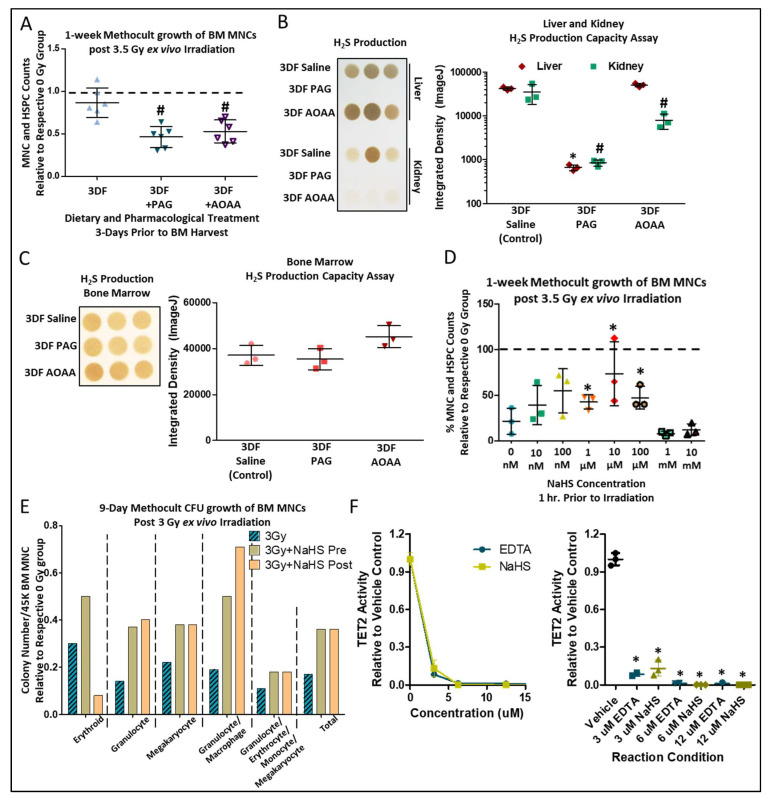
**Pharmacological inhibition of H_2_S producing enzymes dampens fasting induced radio-protection**. (**A**) 1-week Methocult growth counts of BM MNCs and HSPCs normalized to respective 0 Gy controls after *ex vivo* 3.5 Gy irradiation of BM taken from mice under 3DF ± concurrent with *in vivo* propargylglycine (PAG) or amino-oxyacetate (AOAA) administration prior to BM harvest, *n* = 3 mice/group, 6 replicates per condition. Asterisk indicates the significance of the difference between AL and 3DF, and pound indicates the significance of the difference between 3DF and 3DF +PAG/AOAA; */^#^ *p* < 0.05. (**B**,**C**) Lead acetate/lead sulfide H_2_S production capacity assays and their subsequent quantification from the liver and kidney (**B**) or bone marrow (**C**) in mice under 3DF ± PAG/AOAA. *n* = 3 mice/group, asterisk indicates the significance of the difference in liver between control and +PAG/AOAA, and pound indicates the significance of the difference in kidney between control and +PAG/AOAA; */^#^ *p* < 0.05. (**D**) Cell counts after 10-day growth in Methocult of BM after 3.5 Gy *ex vivo* irradiation relative to non-irradiated cells following 1-h incubation with various doses of NaHS (a sulfide generating salt), *n* = 3/group. Asterisks indicate the significance of the difference compared to non-NaHS treatment, *p < 0.05. (**E**) Colony forming unit (CFU) counts from 3 Gy irradiated bone marrow relative to respective 0 Gy controls, with pre-or post-irradiation 10 µM NaHS incubation. (**F**) Recombinant TET2^CD^ protein was incubated with increasing concentrations of NaHS in vitro followed by TET2 activity-based 5hmC ELISA. The left graph displays the data as an X-Y plot, while the right graph displays the data as a column plot for ease of group visualization. Asterisks indicate the significance of the difference compared to the Vehicle control group; * *p* < 0.05, *n* = 3 for the Vehicle Control and NaHS groups. Data are presented as means ± SD.

All animal experiments were performed with approval from Harvard Medical Area Animal Care and Use Committee (IACUC) and the Cleveland Clinic Lerner Research Institute IACUC. Unless otherwise noted, mice were maintained under standard group housing and husbandry conditions with no more than 4 adult mice per cage with initial *ad libitum* access to drinking water and food (Purina 5058), and kept on 12-h light/12-h dark cycles with temperatures between 20–23 °C and 30–70% relative humidity. Male C57BL/6J-DBA/2J F1 hybrid mice (Jackson Laboratories) aged 9–12 weeks were used in Figure 1 and Figure 2, unless otherwise indicated. Female C57BL/6J-DBA/2J F1 hybrid mice (Jackson Laboratories) aged 12–13 weeks of age were used in Figure 3 and Figure 4. In Figure 4E, the bone marrow tested was obtained from a 4-month male C57BL/6 mouse (Jackson Laboratories).

### 2.2. Dietary Preconditioning

Experimental diets are based on a powdered D12450B formula (Research Diets) and contain 18% of calories from hydrolyzed casein protein (Research Diets) and/or individual crystalline amino acids (Ajinomoto), 10% from fat, and 72% from carbohydrates. The food was prepared by rapidly mixing the powdered diet in a 1:1 weight/weight ratio with a warm 2% agar/water solution and allowing the food to cool and form a gel-like solid. This gel-like solid food provides benefits as it is enables for accurate monitoring of the amount eaten, prevents a dominant mouse from hoarding large pieces of food, and facilitates the ease of uniformly mixing and supplementing additional nutrients such as N-acetyl-L-cysteine (NAC), L-methionine, and L-cysteine. For these supplementations into the 50% restricted animal’s diets, NAC (Sigma) added to the food and water equated to approximately 600 mg/kg/day per mouse, L-methionine was supplemented from 2.5 g/kg food in the AL diet to 5 g/kg food in the 50% CR diet, and L-cysteine was supplemented from 2 g/kg food in the AL diet to 4 g/kg food in the 50% CR diet. Thus, in these supplemented diet groups, the total sulfur amino acid content consumed in the 50% CR group is approximately the same as the AL group. After removing the standard facility chow (Purina 5058), animals were given the experimental semi-solid diets and food intake was monitored for several days to determine the correct amount to feed the animals for one week to obtain *ad libitum* or 50% restricted intake prior to animal exposure to radiation or tissue harvest. As all food was consumed in the restricted animals’ cages before the next light period, new food was replaced just before the start of the following day dark cycle between 6 pm and 7 pm to avoid disrupting normal eating patterns and circadian rhythms. Animals that were fasted for 3-days were placed in new cages without food for three days prior to irradiation, with PAG or AOAA administered via IP injection (10 mg/kg a day) during fasting. Mice were given free access to drinking water regardless of diet, and returned to facility chow AL post-irradiation. 

### 2.3. Bone Marrow Isolation and Counting of Bone Marrow Mononuclear Cells

Mice were euthanized via isoflurane overdose and cervical dislocation. Bone marrow from the femur and tibia was immediately flushed with a chilled phosphate-buffered saline (PBS) solution containing 3% fetal bovine serum and 1 mM EDTA and kept on ice. Cells were pelleted by centrifugation and resuspended in 1 mL of the above solution. An aliquot was taken for manual MNC counts using a hemocytometer and microscope after treating it with a 3% Acetic Acid/Methylene Blue solution (StemCell Technologies, Cambridge, MA, USA). After counting, cells were used for *ex vivo* irradiation and/or culture in MethoCult M3434 (StemCell Technologies). Pretreatment of cells with NaHS was accomplished by incubating the flushed BM with 0–10 mM NaHS (SigmaAldrich Inc., St. Louis, MO, USA) in chilled cell suspension buffer one hour prior to *ex vivo* irradiation. For the data obtained in Figure 4E, the BM was flushed and counted in the cell suspension solution, which contains 3% FBS and 1 mM EDTA in PBS. The BM cells were equally distributed (5 × 10^5^) to the sample tubes, followed by irradiation at 0 and 3 Gy. NaHS was given to the cells at 10 µM prior to or after the irradiation treatment for 1 h. After, the cells were washed and plated into MethoCult M3434 (StemCell Technologies) at a density of 500 cells/mL. The cells were cultured in a 5% CO_2_ incubator and counted for colony-forming units (CFUs) after 9 days of culture by visual examination on the size, shape, and color of the colonies as recommended by the manufacturer StemCell Technologies in their technical manual “Mouse Colony-Forming Unite (CFU) Assays Using MethoCult”. All experiments were performed in chilled solutions.

### 2.4. Animal and Bone Marrow Irradiation

After the dietary intervention, single exposure total body irradiation was performed on live, unanesthetized mice at a dose rate of approximately 2 Gy/min on a rotating platform in a Cesium-137 irradiator (Shepherd and Associates). Non-lethal doses administered were between 5.5–7.7 Gy, and lethal dose was 10 Gy, as noted. After irradiation, all mice were placed back into their cages, allowed to recover with *ad libitum* access to the Purina 5058 chow, and then were analyzed at the given time-points for signs of tissue damage, bone marrow cellularity, bone marrow regeneration, and/or survival. For *ex vivo* irradiation, flushed bone marrow in the cell suspension buffer was irradiated in 1.5 mL test tubes on a rotating platform in a Cesium-137 irradiator (Shepherd and Associates) for a total dose of 0 Gy (control non-irradiated) or 3 to 3.5 Gy (irradiated). After irradiation, an equal number of cells were plated into Methocult GF M3434 (StemCell Technologies, Cambridge, MA, USA) and grown for 1–2 weeks in a cell culture incubator at 37 °C in 5% O_2_ and 5% CO_2_. After this growth period, the total colonies were counted while viewing the cell culture plates with a microscope and/or the total cells were counted by adding 1 mL of PBS to the Methocult culture in order to decrease the viscosity, transferring the solution to a 1.5 mL centrifuge tube, briefly spinning down to pellet cells, resuspending in 1 mL PBS, treating an aliquot with 3% Acetic Acid with Methylene Blue solution (StemCell Technologies), and counting the total MNCs with a hemocytometer and microscope. Counts were normalized to the respective non-irradiated control or the AL control group as noted in the figures and legends.

### 2.5. Detection and Analysis of Tissue Damage and Physiological Status

Damage to bone marrow cellularity was detected and visualized by harvesting one of the hind limbs (femur and/or tibia) at timepoints noted in the figures, clearing soft tissue away from the femur, fixing the femur in 4% paraformaldehyde solution in PBS (Santa-Cruz), paraffin embedding, sectioning, and staining with hematoxylin and eosin (H&E staining), followed by visualization under a microscope at 4×, 10×, 20×, or 40× magnification as noted in the figures and legends. H&E staining provides a pink stained section (eosin) for the bone/extracellular matrix, while the purple (hematoxylin) region is the bone marrow (cell nuclei). Lack of the purple (hematoxylin) color is indicative of bone marrow ablation as it is loss of nucleus containing cellularity, while red blood cells lacking a nucleus will stain pink or red and adipose cells create voids in the purple staining. Images presented are representative images taken from each animal and condition. Bone marrow damage was assessed blinded by members of the Dana Farber/Harvard Rodent Histopathology Core. Digital images of euthanized mice were taken for visualization of fur/hair graying and analyzed for mean gray value in ImageJ by importing the original photograph, splitting the channels to get grayscale, subtracting background with rolling ball 50 pixels, and using the tool for analyzing mean gray value on the selected dorsal side of the mice between the back of the ears to the start of the hind limbs. Examination of white and red blood cell parameters in whole blood were performed by collecting fresh blood from the tail vein via heparin coated microhematocrit capillary tubes (VWR) and mixing with 5 µL of anticoagulant citrate-dextrose solution (Sigma) in a 1.5 mL tube prior to running the samples on a Hemavet multispecies hematology analyzer (Drew Scientific). Serum lactate dehydrogenase (LDH) levels/activity were measured from flash frozen serum obtained at the indicated times pre- and post-irradiation via kinetic analysis in a 96-well format on a BioTek microplate reader using the lactate dehydrogenase activity assay kit (Pointe Scientific). Body mass was measured with a digital scale, while body mass composition (lean mass and fat mass) was analyzed via EchoMRI (EchoMRI LLC, Houston, TX, USA).

### 2.6. TET2 Activity Assays

TET2 activity *in vitro* from purified recombinant TET2 via the 5 hmC ELISA in the presence of increasing NaHS or EDTA concentrations was performed as previously described [31]. 

### 2.7. H_2_S Production Assay

H_2_S production capacity from tissues and serum was performed via the lead acetate/lead sulfide method [32]. Briefly, tissues were homogenized and lysed in Passive Lysis Buffer (Promega) using flash freezing/thawing in liquid nitrogen to crack the cellular membranes. After clearing debris via centrifugation, protein concentrations were determined and normalized via the Pierce BCA assay (ThermoFisher Scientific, Waltham, MA, USA). Equal amounts of protein per sample were added to a 150 µL reaction in 96-well plate format containing 10 mM L-cysteine (Sigma) and 1 mM Pyridoxal 5′-phosphate hydrate (Sigma) in PBS. A lead acetate embedded filter paper was placed above the wells and incubated at 37 °C until detectable, but not saturated, and lead sulfide circles appeared above the wells. The lead sulfide circles were analyzed for integrated density using the IntDen function in ImageJ as previously described [26].

### 2.8. Data Quantification and Statistical Analysis

Data were compiled and analyzed in Microsoft Excel and/or GraphPad Prism using Student’s *t* tests to compare values between two specific groups or a one-sample t test when comparing means to a value of 1 or 100 when data were normalized to the average value of the respective control or unirradiated counterpart group. In analyzing body mass, fat mass, lean mass, and food consumed in Figure 3, *t*-tests with pairing at each daily time point were performed. When comparing more than two groups, a one-way ANOVA with Bonferroni correction was performed. In all cases, a *p*-value of 0.05 or less was deemed as statistically significant. Kaplan–Meier survival plot of lethally irradiated mice was performed using GraphPad Prism, with a Mantel–Cox Log-rank test to determine *p*-value. Data are displayed as means ± standard deviation (SD). All statistical analyses were run with *n* = 3 to 11 individual animals per group as noted in the figure legends, with technical duplicates or triplicates run from cells and tissues derived from each animal.

## 3. Results

### 3.1. Short-Term CR Protects against Sub-Lethal γ-Irradiation Induced Damage In Vivo

Exposure to total body irradiation (TBI) above 3 Gy for humans [33] and 10 Gy for most mouse strains [34] is lethal within days to weeks. However, sub-lethal TBI accelerates aging and permanently damages multiple tissues. We examined how short-term CR in young adult mice prior to a variety of sub-lethal TBI exposures impacted skin, blood, and BM. Mice were fed a complete diet either *ad libitum* (AL) or restricted to 50% of AL intake (caloric restriction; CR) one week prior to sub-lethal TBI, and were then fed AL after irradiation. Serum lactic acid dehydrogenase (LDH), a marker for global tissue damage, equally increased in AL and CR fed mice within 1–6 h post 8.5 Gy TBI and returned near baseline within 24 h (Figure 1A). Twenty one weeks after 7.7 Gy TBI, heterogeneous graying of the fur was increased (Figure 1B) and circulating total white blood cells (WBC), lymphocytes (Figure 1C), and hematocrit (Figure 1D) decreased relative to unirradiated controls. Importantly, these negative effects on heterogeneous graying, WBC, and lymphocytes, but not hematocrit, were largely ameliorated by CR prior to irradiation.

As these results in the skin and blood indicate possible involvements of stem cell and/or hematopoietic systems, we next examined acute changes in BM cellularity and regrowth as a function of radiation and diet. Seven days post 6.5 Gy TBI, numbers of flushed hindlimb BM mononuclear cells (MNC) were decreased in both AL and CR mice compared to non-irradiated controls, with a trend towards increased MNC counts in CR mice (Figure 1E). By day 18 post TBI, the BM MNC counts were higher in the CR mice compared to AL mice (Figure 1E). These changes in BM cellularity were visually detected in fixed and H&E stained hind limb bones, showing an overall reduction in hematoxylin-stain nucleus containing cells and an increase in nucleus-free eosin-stained cells post-irradiation that is more pronounced in AL fed mice compared to CR red mice as seen on day 7 post-TBI (Figure 1F,G) and day 18 post-TBI (Figure 1H). Thus, 50% CR for one week prior to sub-lethal TBI offers a measurable benefit for regrowth in BM and hematopoietic systems.

### 3.2. Sulfur Amino Acid Supplementation Suppresses the Radio-Protective Benefits of CR

Supplementation of the restricted diet with sulfur amino acids (SAA) L-methionine (Met) and L-cysteine (Cys), N-acetyl cysteine (NAC), or homocysteine abrogates the stress-resistance and longevity benefits of CR [30,35]. To test if short-term CR-mediated radio-resistance/resilience is also abrogated by SAA supplementation, NAC was administered prior to irradiation to the AL and CR fed mice (Figure 2A). NAC supplementation did not impact diet induced weight changes before and after TBI (Figure 2B). However, CR+NAC prior to TBI reduced the radio-protective effect of CR, resulting in reduced numbers of flushed hind limb BM MNCs (Figure 2C) and decreased hindlimb BM cellularity and hematoxylin staining of nucleus containing cells (Figure 2D,E) 1 week after radiation when compared to the CR alone. As depicted in Figure 2A, the cell autonomy of this increased proliferative capacity was confirmed by plating equal numbers of BM MNCs 7 days post TBI into semi-solid Methocult media supporting the growth of BM MNCs and HSPCs [36]. Over a 1 week period, *ex vivo* growth of MNCs from the CR+NAC group was decreased relative to the CR group (Figure 2F).

We next tested whether CR-mediated radio-resistance/resilience requires intact BM *in vivo,* or if it is a cell intrinsic property of BM observable *ex vivo*. To this end, BM MNCs were flushed from mouse femurs after the 7 day preconditioning period and then irradiated 3 to 3.5 Gy *ex vivo* prior to plating of equal numbers into Methocult as depicted in Figure 2G, with 0 Gy groups serving as controls. After 1 week, proliferation and/or growth in Methocult was not largely different between diet groups in the 0 Gy controls (Figure 2H,I), but it was greater in irradiated BM from CR mice than from AL mice, and this effect was lost upon supplementation with either NAC or Met&Cys as depicted by absolute cell counts (Figure 2H,I) and when plotted as a ratio to the respective 0 Gy control group (Figure 2J,K). Taken together, these data are consistent with cell autonomous radioprotection *ex vivo* when induced *in vivo* by CR and abrogated by dietary SAA supplementation.

### 3.3. CGL Activity is Necessary, While H_2_S is Sufficient, for Short-Term CR-Mediated BM Radioprotection

We previously reported increased CGL expression correlates with increased endogenous H_2_S production capacity and is required for DR-mediated protection from hepatic ischemia reperfusion injury (IRI) [30], the growth of blood vessels in skeletal muscle [28], maintaining proper IGF-1 and thyroid hormonal response to fasting [37], protection against vein graft disease [12], and expanding tissue-specific protein persulfidomes [38]. Exogenous H_2_S not only protects against multi-tissue IRI [39,40,41,42], but protects against radiation-induced myelosuppression [43] and cell death [44]. We thus examined the requirement for sulfur amino acid metabolism via the transsulfuration pathway enzyme CGL, along with H_2_S itself, in DR-mediated radioprotection in BM and hematopoietic systems. To do so, we turned to 3-day fasting (3DF) as a short-term CR preconditioning regimen in combination with small molecule inhibitors of CGL. Fasting 2 to 3 days prevents hepatic and renal ischemic reperfusion injury [45]. The 3DF conferred radioprotection to BM after exposure to non-lethal TBI as determined from *in vivo* cellularity in H&E stained hind limb bones (Figure 3A), cell counts in flushed hind limb bones (Figure 3B), and *ex vivo* growth (Figure 3C). Notably, a 3DF prior to lethal 10 Gy TBI extended mean and maximum survival times by ~12% (Figure 3D). Concurrent with the increased survival were rapid drops and then gains in total body mass (Figure 3E), fat mass (Figure 3F), and lean mass (Figure 3G) prior to and after TBI, respectively, that corresponded with increased post TBI food consumption in the 3DF group compared to the AL group (Figure 3H). 

*In vivo* administration of propargylglycine (PAG), a suicide inhibitor of CGL, or amino-oxyacetate (AOAA), which acts as an inhibitor of CGL as well as other enzymatic and non-enzymatic reactions utilizing the co-factor vitamin B_6_ for H_2_S generation [46,47], concurrent with 3DF, blunted radio-protective effects in BM cells irradiated *ex vivo* (Figure 4A). PAG inhibited H_2_S production in both the liver and kidney (Figure 4B) and AOAA inhibited H_2_S production in the kidney (Figure 4B). However, H_2_S production in total BM was not changed in relation to PAG or AOAA treatment (Figure 4C), suggesting a systemic DR-induced CGL/H_2_S effect, possibly emanating from the liver or kidney, that provides lasting radioprotection to the BM. This aligns with the data we recently reported on brain and muscle H_2_S-induced persulfidation increases in WT mice, but not CGL KO mice, under CR despite there being no detectable genotype- or diet-induced changes in H_2_S production in the brain or muscle [38]. Likewise, we also found renal, but not brain, H_2_S production capacity had strong correlative ties to cognitive outcomes in aged mice [48,49]. Thus, H_2_S produced in the liver and/or kidney via CGL potentially acts as a circulating gasotransmitter that imparts its function locally as well as systemically, including in the BM.

CGL is a multi-functional enzyme that could confer radio-resistance/resilience via increased H_2_S production, or in other non-mutually exclusive ways, for example via cysteine production with indirect effects on glutathione or taurine biosynthesis, all of which have been shown to offer some form of radioprotection [50,51,52]. We tested the sufficiency of H_2_S to confer radio-resistance/resilience to BM MNCs. Pretreatment for 1 h with NaHS, a sulfide/H_2_S generating salt, up to 100 µM prior to irradiation improved growth in Methocult after *ex vivo* irradiation, with beneficial/physiological [53] doses between 0.1–100 µM and lethal doses at or above 1 mM (Figure 4D). In further examining the subtypes of colony growth in Methocult, we determined through visual colony forming unit (CFU) assay that both pretreatment and posttreatment of BM with 10 µM NaHS for 1 h enhanced granulocyte, megakaryocyte, and macrophage CFUs (Figure 4E). However, only NaHS pretreatment, not posttreatment, improved erythroid CFUs (Figure 4E). Ten eleven translocation 2 (TET2) is a DNA dioxygenase that regulates HSPC expansion and differentiation via CpG demethylation, with loss of function or enzymatic inhibition leading to HSPC clonal expansion [31]. Increasing NaHS concentrations incubated with recombinant TET2^CD^ in vitro inhibited TET2 activity as detected by 5hmC assays to similar extents as EDTA, and provided an approximate IC_50_ of 2.9 µM (Figure 4F, left and right). Taken together, these data show that short-term DR protects BM from ionizing radiation in a cell autonomous manner that potentially relies on H_2_S-generating enzymes and the inhibition of TET2 enzymatic activity. 

## 4. Discussion

**Figure 5:**
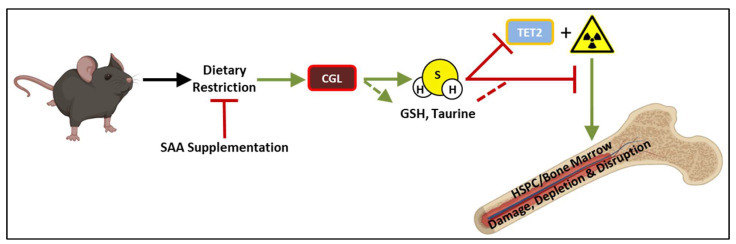
Overarching model of nutritional and molecular requirements for dietary restriction enhanced bone marrow and HSPC regeneration following exposure to ionizing radiation. Short-term (3–7 day) dietary restriction preconditioning, and specifically caloric restriction or fasting, prior to ionizing irradiation *in vivo* or *ex vivo* promoted regrowth of bone marrow. Dietary supplementation of sulfur amino acids (SAAs) and/or pharmacological inhibition of the transsulfuration enzyme CGL concurrent with dietary restriction abrogated its protective effect on the bone marrow. Conversely, exogenous application of hydrogen sulfide (H_2_S) on bone marrow prior to irradiation promoted regrowth *ex vivo*, and corresponds to the inhibition of TET2 providing a potential non-mutually exclusive mechanism of action for H_2_S outside its antioxidant and persulfidation activities. SAA: sulfur amino acids, CGL: cystathionine γ-lyase, GSH: glutathione, HSPC: hematopoietic stem and progenitor cell, H_2_S: hydrogen sulfide, TET2: Ten eleven translocation 2.

Previously, it was demonstrated that short-term DR regimens involving CR, fasting, or protein/essential amino acid restriction increase resilience and recovery in preclinical models of sterile inflammation including ischemia reperfusion injury in solid organs [25,30] or intimal hyperplasia upon vascular occlusion [54]. Here, we extended the ability of DR, in the forms of CR and fasting, to precondition against a very different types of clinically relevant stress, ionizing radiation, and in a different tissue/cell type—the bone marrow. As in CR-mediated resistance to hepatic ischemia reperfusion injury [30], the addition of sulfur amino acids here also abrogated protection, consistent with a role for sulfur amino acid metabolism and the TSP enzyme CGL, confirming the protective benefits of DR. As several sulfur-containing downstream products of CGL activity, such as glutathione and taurine, have radio-protective and radio-recovery potentials [50,55], we showed specifically that the exogenous addition of another one of its products, H_2_S, was sufficient to drive the radioprotection of BM cells *ex vivo* in a dose dependent manner. These findings are summarized in the model depicted in Figure 5.

While our study suggests the importance of sulfur amino acid metabolism and/or sensing in CR-mediated radioresistance, the mechanism by which reduced SAAs and/or H_2_S confer protection remains unknown. Ionizing radiation damages cells directly via injury to DNA and other biological macromolecules, as well as indirectly through increased hydroxyl radical and ROS generation from water [56]. These damages elicit pro-apoptotic signaling cascades, particularly in dividing cells, and ultimately result in disturbances in stem cell renewal and differentiation. H_2_S has antioxidant and reducing capabilities [57], which could counteract ROS and block these destructive pathways. Additionally, as we have recently shown the ability of CR and fasting to modify and enhance tissue-specific protein persulfidation (aka sulfhydration) [38], it is possible this posttranslational modification interferes with irreversible oxidation of protein thiol residues resultant to ionizing radiation. In addition to protection against irreversible protein thiol oxidation, protein persulfidation of DNA repair factors could also enhance radio-protection, as it has been shown that persulfidation of MEK1 at cysteine 341 leads to PARP-1 activation and enhances DNA damage repair [58].

Diet, the hypothalamic-pituitary axis, and CGL/H_2_S are tightly linked and regulate each other. Growth hormones (GH) and thyroid hormones (TH) repress hepatic CGL expression and H_2_S production, while endogenous H_2_S production downregulates GH, TH, and IGF-1 signaling [37,59]. CR-mediated downregulation of IGF-1 is required for diet-induced BM HSC stress resistance, self-renewal, and maintaining proper lineage balance when exposed to genotoxic and replicative stressors [18]. We previously reported that dietary restriction, specifically fasting, decreased circulating IGF-1 levels in a CGL-dependent manner [37]. Thus, increased CGL/H_2_S under CR or fasting concurrent with a reduction in SAAs may serve as triggers to suppress global and/or BM specific IGF-1 signaling, and this serves as an additional mechanism of action for radio-protection and/or regrowth of the BM.

Interestingly, exogenous H_2_S delivered as NaHS *in vivo* can also confer protection against radiation by preventing myelosuppression, platelet loss, and death [43]. Conversely, pretreatment with chemical inhibitors of CGL increases lethal radiation-induced bystander effects in cells by augmenting Bcl-2/Bax and caspase-3 mediated apoptosis, and this is ultimately reversed with NaHS treatment [60]. These results mirror our current observations with BM cells presenting with enhanced *ex vivo* Methocult hematopoietic regrowth post irradiation when preconditioned with NaHS in a dose dependent manner. While we have focused this study on hematopoietic bone marrow regrowth, interestingly, it was previously shown that enhanced transsulfuration activity and H_2_S generation also maintain bone marrow mesenchymal stem cell function and bone homeostasis [61]. Outside of the bone marrow, a recent study showed that multiple cell types produce H_2_S when exposed to various forms of genotoxic stress, and this production provides a protective barrier against cell death and senescence [62]. This response has benefits in promoting non-cancerous cell survival against genotoxic stress often seen during cancer therapy, with the hopes that cancerous cells do not benefit from the H_2_S. Thus, enhanced sulfur amino acid metabolism and H_2_S generation/signaling prior to and/or during genotoxic stress appear to be crucial for cellular survival and regrowth.

The relationship between CGL expression and leukemogenesis has been known for decades [63], resulting in cysteine auxotrophy [64]. Decreased CGL expression may be a signature for poor prognosis in hematological oncology cases, and suggests its role as a tumor suppressor gene. Conversely, CGL and/or H_2_S are beneficial to normal BM. Our molecular mechanistic hypothesis is that enhanced levels of circulating H_2_S mediated by dietary restriction lower TET2 activity in BM MNC and HSPCs and promote their expansion. With decreased H_2_S production and signaling during aging [65], TET2 activity would thus increase, leading to lower self-renewal of wildtype TET2 HSPCs compared to their pro-cancerous loss-of function *TET2* mutants [31,66]. Thus, in addition to providing BM radiation resistance, CR or CR mimetics may provide an advantage for non-cancerous TET2 WT BM HSPCs to compete against enzyme inactive *TET2* mutant HSPCs, potentially limiting aplastic anemia and/or myelodysplastic syndromes. 

Thus, why does supplementation of SAAs during DR abrogate protection against bone marrow damage and hinder repopulation post irradiation? During the 50% CR precondition we provided to mice, it should be noted that this results in the reduction of SAAs by 50%. As reduction in overall protein and SAA intake act as strong drivers of resilience and the integrated stress response via GCN2/ATF4-dependent mechanisms [67,68], it is plausible the addback of SAAs blunts this response. GCN2/ATF4 regulates CGL and H_2_S production in response to SAA restriction [26,28,69] and is critical for survival and proliferation of primary bone marrow cells [70]. Thus, having this integrated stress signaling cascade primed by DR appears to be crucial for radioresistance. Similar phenomena have been reported regarding SAA supplementation abrogating the benefits of DR, such that lifespan is diminished in rodents [35], and the liver and heart are sensitized to ischemic reperfusion injury [30,71]. Likewise, supplementation of just NAC or cysteine is sufficient to reverse DR-mediated stress resistance in mice as reported here and previously [30], and it can also reverse the anti-obesity effects of methionine restriction [72,73].

Although therapeutic DR is safe and effective in animal models and in humans under closely supervised conditions [74,75], the extent of 50% daily restriction utilized in this study may be difficult for the majority of individuals and/or potentially dangerous for those with certain underlying metabolic disorders and nutritional requirements [76,77]. However, it was recently reported that reducing daily caloric intake by just 14% in humans over a 2 year span was sufficient to drive CR-induced transcriptional reprogramming related to bioenergetics, anti-inflammatory responses, and longevity [78]. Thus, such severe restriction of total food intakes by 50% may not be necessary to drive radioresistance in humans. Likewise, *ad libitum* isocaloric approaches that focus on just short-term SAA or essential amino acid restriction may be a safer alternative with greater compliance. In our study, it is important to note the utilization of 50% CR also results in 50% restriction of the SAAs methionine and cysteine in that 1-week preconditioning window. Richie and colleagues reported the average intake of SAAs for adults in the USA being 2.83 ± 0.89 g/day resulting in 39.2 ± 18.1 mg/kg/day consumption [79]. This level is 2.5-fold higher than the Estimated Average Requirement of 15 mg/kg/day [79], and thus, it puts to question just how much lowering of SAAs in the human diet would be needed to confer radioprotection, and for how long? Would it have to be 2.5-fold lower than the current levels ingested, or somewhere in the range of 7.5 mg/kg/day equated to 50% of the Estimated Average Requirement? Furthermore, regardless of the levels and duration of decreased SAAs, what palatable natural or synthetic dietary formulation would successfully and safely provide such reduction? When comparing the mean content (g amino acid/100 g food) of SAAs in animal based diets to vegan diets in humans, it appears SAA intake is already reduced from ~0.7 down to ~0.2, or approximately 3.5-fold in the vegan diets [80]. Thus, a switch to a plant-based diet prior to or concurrent with genotoxic stress may provide some benefit to the hematopoietic system. Alternatively, DR mimetics such as metformin can also increase H_2_S levels in rodent tissues [81] and also attenuate ionizing radiation-induced BM HSC injury [82], although whether the radioprotective effects are dependent on enhanced H_2_S remains undetermined. Future studies are required to better understand the mechanism of H_2_S action in radioresistance, the stem cell and progenitor cell populations most impacted by diet and H_2_S, and practical approaches to translate these findings to the clinic.

As our study focused more so on the nutritional and metabolic regulation of bone marrow regrowth post irradiation, it has several technical, mechanistic, and cellular-level limitations. First, our study does not go into the phenotypic analysis (myeloid, lymphoid, etc.) in great detail for the reconstituted cellularity of the bone marrow. While we do provide data on circulating white blood cell and red blood cell parameters in the aged/irradiated mice post TBI, we do not go into the cell and lineage types found directly in the damaged and re-growing bone marrow. This is a worthwhile investigation and topic of further research examining more so the cell intrinsic and cell extrinsic genetic requirements for CGL in driving the radio-protective effects of DR in the BM niche. Second, we do not directly investigate the levels of cellular DNA and macromolecular damage induced by irradiation and how DR impacts these endpoints. The enhanced repopulation of bone marrow *in vivo* and *ex vivo* that we detected could be due to decreased initial damage, enhanced repair, diminished localized inflammation, or a combination of these and other stem and progenitor proliferation regulators. Likewise, as the study focused primarily on both MNCs and HSPCs combined, it cannot accurately convey the function of these cells and their respective subtypes in the post-irradiation bone marrow niche and/or systemically. Lastly, while the study delved primarily into the bone marrow response, we feel that the novel qualitative results on fur graying in Figure 1, despite our lack of mechanistic underpinning, are still worthy of inclusion as a unique aspect of diet mediated protection against irradiation induced changes that also mimic aging-related declines in health and physiology.

Overall, our preclinical results suggest that dietary reduction in calories and SAAs, increasing endogenous H_2_S production capacity, and/or safely administered H_2_S exogenously present as therapeutic avenues to preserve BM homeostasis for patients undergoing genotoxic radio- or chemo-therapies. Reducing calories or SAA intake by 25–50% or switching to a low protein vegan diet that is naturally low in SAAs [80,83] for BM donors in the days just prior to tissue harvest, or boosting H_2_S levels exogenously to the donor BM tissue, may be feasible approaches to promote improved transplantation outcomes. Similar applications using H_2_S to improve organ transplantation success have been shown preclinically for the kidney and heart [84,85]. Future translational studies will be required to determine safety and efficacy of CR or SAA restriction vs. supplementation with H_2_S donors for improved bone marrow related outcomes. 

## Data Availability

Data supporting the reported results and conclusions can be found in the figures. Requests for additional research materials and protocols will be fulfilled from the corresponding author.

## Abbreviations

AL: Ad libitum, AOAA: amino-oxyacetate, BM: bone marrow, CGL: cystathionine γ-lyase (aka CSE, CTH), CR: calorie restriction, Cys: L-cysteine, DR: dietary restriction, GH: growth hormone, H_2_S: hydrogen sulfide, HSC: hematopoietic stem cell, HSPC: hematopoietic stem and progenitor cell, IR: ionizing radiation, KO: knockout, Met: L-methionine, MNC: mononuclear cell, NAC: N-acetyl cysteine, NaHS: sodium hydrosulfide, PAG: propargylglycine, RBS: red blood cell, SAA: sulfur amino acid, TBI: total body irradiation, TET2: Ten eleven translocation 2, TH: thyroid hormone, TSP: transsulfuration pathway, TNFα: tissue necrosis factor α, WBC: white blood cell, WT: wildtype.
